# Dual Binding Site and Selective Acetylcholinesterase Inhibitors Derived from Integrated Pharmacophore Models and Sequential Virtual Screening

**DOI:** 10.1155/2014/291214

**Published:** 2014-06-25

**Authors:** Shikhar Gupta, C. Gopi Mohan

**Affiliations:** ^1^Department of Pharmacoinformatics, National Institute of Pharmaceutical Education and Research (NIPER), Sector 67, S.A.S.Nagar, Punjab 160 062, India; ^2^Amrita Centre for Nanosciences and Molecular Medicine (ACNSMM), Amrita Institute of Medical Sciences and Research Centre, Amrita Vishwa Vidyapeetham University, Ponekkara, Kochi, Kerala State 682 041, India

## Abstract

In this study, we have employed* in silico* methodology combining double pharmacophore based screening, molecular docking, and ADME/T filtering to identify dual binding site acetylcholinesterase inhibitors that can preferentially inhibit acetylcholinesterase and simultaneously inhibit the butyrylcholinesterase also but in the lesser extent than acetylcholinesterase. 3D-pharmacophore models of AChE and BuChE enzyme inhibitors have been developed from xanthostigmine derivatives through HypoGen and validated using test set, Fischer's randomization technique. The best acetylcholinesterase and butyrylcholinesterase inhibitors pharmacophore hypotheses Hypo1_A and Hypo1_B, with high correlation coefficient of 0.96 and 0.94, respectively, were used as 3D query for screening the Zinc database. The screened hits were then subjected to the ADME/T and molecular docking study to prioritise the compounds. Finally, 18 compounds were identified as potential leads against AChE enzyme, showing good predicted activities and promising ADME/T properties.

## 1. Introduction

Alzheimer's disease (AD) is a neurodegenerative disease involving impairment of cognitive function with both genetic and nongenetic causes, which is characterized by a loss of basal forebrain cholinergic neurons and reduced level of neurotransmitter acetylcholine (ACh) in hippocampal and cortical levels, leading to severe memory and learning deficits [1]. AD is caused by a progressive and specific degeneration of neurons; with extracellular deposition of *β*-amyloid plaques, intracellular deposition of neurofibrillary tangles, which lead to neurotoxicity and synaptic loss being hallmarks of the disease. Enormous research effort has been made to understand the molecular pathogenesis of AD from the last few decades. However, the only symptomatic treatment based on the cholinergic hypothesis targeting acetylcholinesterase (AChE) (EC 3.1.1.7) enzyme is one of the major therapeutic strategies adopted for symptomatic relief on AD [2]. This hypothesis is proven to be successful today by the effective use of cholinesterase inhibitors such as tacrine, rivastigmine, donepezil, and galanthamine to augment surviving cholinergic activity for the treatment of mild to moderate AD. However, patient's undergone medications with these inhibitors had shown only modest recovery with some adverse effects. Many AChEIs also inhibit BChE (EC 3.1.1.8), because both AChE and BChE enzymes are found in the central nervous system (CNS). Moreover, AChE and BChE share 65% amino acid sequence homology even though being encoded by different genes on human chromosomes [3]. The BChE inhibition can lead to adverse peripheral side effects [4]. Functionally, both enzymes hydrolyze acetylcholine efficiently but at different rate, that is, at the same temperature and pH, AChE has higher hydrolytic acetylcholine activity than BChE [5]. The role of AChE PAS has been identified in the enhancement of the aggregation of A*β* fragments, which accelerates the assembly of A*β*_1–42_ peptide responsible for neurodegenerative process in AD [6, 7]. It has been shown that AChEIs simultaneously binding to the active and PAS of enzyme (dual-site inhibitor) are responsible for the enhanced binding of the gorge-spanning ligands. AChEIs, donepezil, and decamethonium [8] could prevent the A*β* aggregation apart from its cholinergic activity [9]. Hence, Dual binding site AChEIs have been currently recognized as a new strategy to identify the more efficacious and promising anti-Alzheimer's candidates to positively modify the course of the AD.

The physiological role of BChE is still unclear. Moreover, BChE did not affect amyloid formation because three aromatic residues of the AChE PAS are missing in the PAS of BChE [15]. Hence, the PAS of BChE had weaker affinity than AChE, which mediates substrate activation. However, BChE may play a compensatory role in the hydrolysis of acetylcholine in brain with degenerative changes. Indeed, AChE activity decreases in certain brain regions as AD progresses, while BChE activity is not affected or even increases, making BChE available in neuritic plaques. Hence, mixed inhibition of AChE/BChE enzymes could lead to an improved AD therapeutic benefit. But, the inhibition of BChE more than the AChE can lead to adverse peripheral side effects. Tacrine, the first FDA approved drug for the treatment of AD, has more activity towards BChE than AChE and is hepatotoxic in nature. While, the bis-7 tacrine a bifunctional (dual binding site AChEI) homodimer of tacrine was found to be 10000 fold more selective and 1000 fold more potent than tacrine for AChE inhibition without having toxic effect [4].

The differences in the enzyme kinetic properties and locations of brain of AChE and BChE have led to the suggestion that, in the normal brain, AChE is the main enzyme responsible for acetylcholine hydrolysis, while BChE plays a supportive functional role [16]. The main difference in the acyl-binding pocket of both these enzymes is that F288 and F290 in AChE were replaced by L286 and V288 of BChE [17]. Therefore, design of dual binding site and selective AChEIs such as donepezil has recently presented a new and potential therapeutic strategic option for the treatment of AD [18, 19]. Recently, our research group identified few potent and selective AChEIs by integrating* in silico* and* in vitro* analysis [20, 21].

Identification of the pharmacophoric features is one of the most important computational approaches in a rational drug design process. 3D-pharmacophore generation is useful for identifying the important pharmacophoric features, which could help in designing new compounds [22–25]. It represents the interaction between a receptor and a ligand and has been successfully applied for 3D search of large small compounds, also termed as virtual screening (VS) of chemical databases [26, 27]. It is one of the most promising computational methods to reduce unwanted compounds at the early stage of the drug discovery process [28–30]. However, the available databases become larger and their experimental testing is very expensive. Therefore, a small subset of the database compounds that are likely to bind with the target was further carried forward for experimental screening. This selection can be performed by VS through small compound databases, fitting a known pharmacophore and/or a 3D structure of the target [31, 32].

In this study, specific 3D-pharmacophore models of AChE and BChE inhibitors have been developed from structurally diverse xanthostigmine derivatives [10], using 3D-pharmacophore generation module in Accelrys Discovery Studio2.5 (DS2.5) [33] software, which is based on HypoRefine algorithm. To identify potent and selective dual binding site AChEIs, it was important to know the chemical requirement of these inhibitors and structural differences between the binding pockets of AChE and BChE, respectively. The main objective of the present work was to identify selective and dual binding site AChEIs by generating and integrating AChE and BChE based pharmacophore models in sequential VS strategy. The screened compounds were then further validated using molecular docking analysis, in order to understand its selectivity as well as the mode of interactions at the dual binding site of the AChE enzyme. To the best of our knowledge, this is the first integrated pharmacophore model based VS approach to identify selective and dual binding site AChEIs. The simultaneous use of pharmacophore based VS, physicochemical screening, and molecular docking is anticipated to make drug discovery more efficient in the hit selection process [34].

## 2. Materials and Methods

### 2.1. Molecular Modeling

All compounds were built using the SYBYL7.1 (Tripos) molecular modeling package installed on a SGI Workstation running IRIX 6.5. Gasteiger-Hückel partial atomic charges [35] were assigned to the compounds and their conformational energy was minimized using the Powell [36] method and the Tripos force field [37] with a convergence criterion for the energy gradient 0.001 kcal/mol/Å.

### 2.2. Selection of Compounds

**Figure 1:**
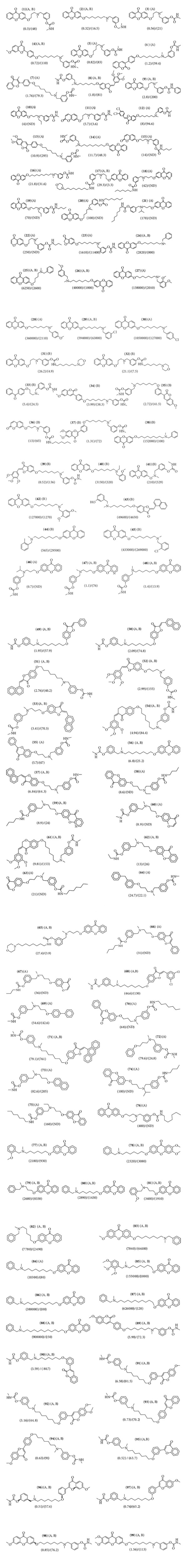
Chemical structures of training set and test set compounds for AChE (A) and BuChE (B) (**1**–**99**) together with their biological activity data IC_50_ (nM) (AChE)/(BuChE), in parentheses [10–14]. ^∗^ND: not determined.

The data set of 99 xanthostigmine derivatives used in the study for the 3D-pharmacophore generation was collected from the literature [10–14]. AChE activity of these inhibitors spans a range of six orders of magnitude from 0.0003 *μ*M to 1050 *μ*M and for BChE inhibitors five orders of magnitude from 0.0033 *μ*M to 269 *μ*M, respectively. These activity values were measured under the same bioassay technique. The dataset is further divided into two different categories in accordance with the AChE and BChE activity values. AChE data set of 95 compounds was divided into a training set of 30 compounds and a test set of 65 compounds, and BChE dataset of 77 compounds was divided into a training set of 26 compounds and a test set of 51 compounds, respectively, by taking into account its structural diversity and activity range. The training set compounds were then used to generate the 3D-pharmacophore models for the respective enzymes, while the test set compounds were used for its model validation. The chemical structures along with their AChE/BChE (A, B) activity (IC_50_) in nM of training set and test set compounds are presented in Figure 1. It is essential to assess the predictive power of the pharmacophore models by using a test set of compounds according to the following criteria. (1) Biological activity values of the test set should span the training set. (2) The biological assay methods for both the training set and the test set should be the same or comparable. (3) The test set should represent a balanced number of both active and inactive compounds for uniform sampling of the data set.

### 2.3. Pharmacophore Models Generation and Validation

All the pharmacophore modeling calculations were carried out by using HypoGen method implemented in DS2.5 software. Multiple acceptable conformations were generated for all AChE and BChE inhibitors of the training set by Cat-Conf program within DS2.5 software. Conformations of all the inhibitors were generated by using the “Best conformer generation” with 20.0 kcal/mol as energy cutoff, and maximum number of conformers was selected 255, while all other parameters were set to default, except the Uncert value of 3.0 for all the compounds. Instead of using just the lowest energy conformation of each compound, all conformational models for each compound in training set were used for pharmacophore hypothesis generation. Selecting the chemical feature is one of the most important steps in generating pharmacophore. Features such as hydrogen-bond donor (HBD), hydrophobic-aromatic (HY-AR), hydrophobic aliphatic (HY-Al), and ring aromatic (RA) were included for AChEIs, while hydrogen-bond acceptor (HBA), hydrogen-bond donor (HBD), hydrophobic aliphatic (HY-Al), hydrophobic-aromatic (HY-AR), and ring aromatic (RA) were selected for BChE inhibitors based on an overview of all the training set compounds for both AChE and BChE. Starting with a training set of 30 AChEIs and 25 BChE inhibitors, pharmacophore models (also called a hypothesis) able to quantitatively correlate the predicted affinities with the corresponding measured values were generated, by using the 3D QSAR pharmacophore generation module within the DS2.5 software. Ten pharmacophore models with significant statistical parameters were generated for both AChE and BChE. The best model was selected on the basis of a high correlation coefficient (*r*), lowest total cost, highest cost difference, and lowest RMSD values. Fixed cost represents a simple model that fits all data perfectly, while null cost presumes that there is no relationship in the data and that the experimental activities are normally distributed around their average value. And total cost sums over error cost, weight cost, and configuration cost. For an expected pharmacophore model, the total cost should be close to the fixed cost, and there should be a significant difference between null and total cost. Further, a value of 40–60 bits for the unit of cost difference implies a 75–90% probability of the correlation between experimental and predicted activities.

Model validation is a critical step in automated pharmacophore generation, especially in those cases where the model has been generated for the purpose of VS of different small compound databases. Two validation procedures were followed, namely, test set prediction method and Cat-Scramble method. Our test set covers the similar structural diversity to the training set in order to ascertain the broadness of pharmacophore predictability. The final pharmacophore models of AChE and BChE were validated by its ability to predict the affinity of test set of 65 AChEIs and 51 BChE inhibitors. The Cat-Scramble validation procedure is a cross-validation based on Fischer's randomization test. The goal of this type of validation is to check whether there is a strong correlation between the chemical structures and the biological activity [38]. This is done by randomizing the activity data associated with the training set compounds, generating pharmacophore hypotheses using the same features and parameters to develop the original pharmacophore models. To achieve the confidence level of 90%, 9 random spreadsheets and for 95% confidence level 19 (random hypotheses) were generated. The importance of the hypotheses was calculated using the following formula:

(1) $$\begin{array}{c} \text{Significance}=\left[1-\left(1+\frac{\text{A}}{\text{B}}\right)\right]\times 100, \end{array}$$

where A, total number of hypotheses having a total cost lower than Hypo A and B, total number of Hypogen runs (initial + random runs). Here, A = 0 and B = (19 + 1), *S* = [1 − ((1 + 0)/(19 + 1))] × 100% = 95%. Overall, we verified that our 3D-QSAR pharmacophore models were predictive not only within the training set compounds but also for the test set compounds, with acceptable errors of value.

### 2.4. Database Preparation and Virtual Screening

**Figure 2:**
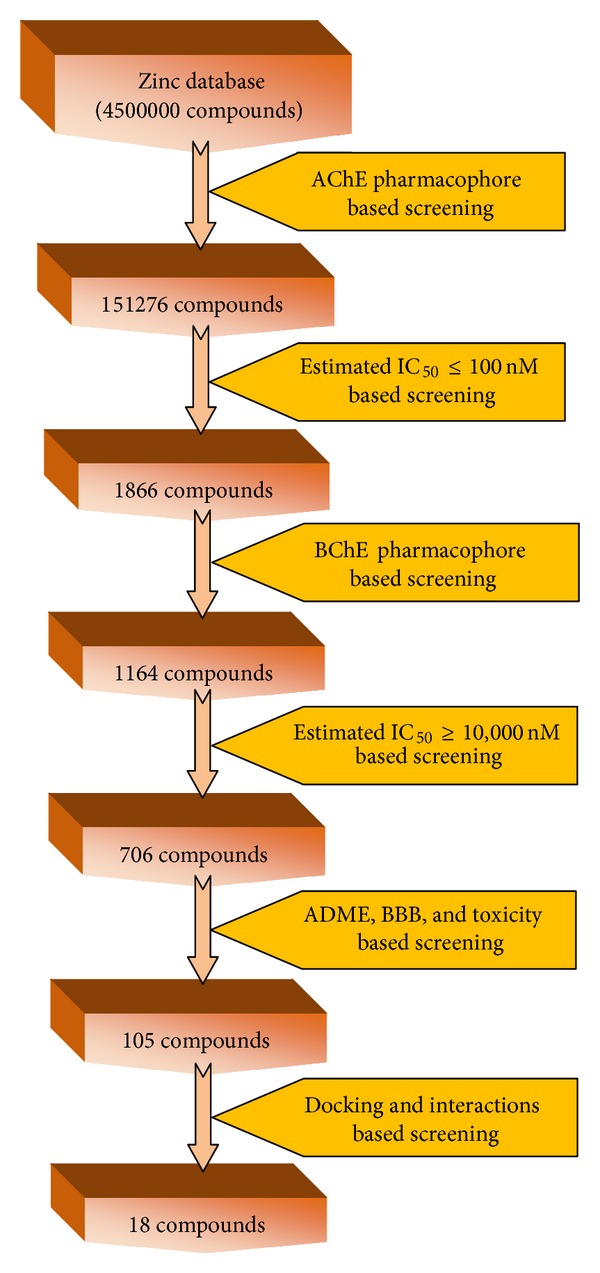
Flow chart showing sequential virtual screening technique used in the present study.

We adopted sequential VS strategies to reduce the search space for potential drug-like candidates by filtering out compounds unlikely to interact with the AChE enzyme. The ZINC [39] database containing ~4500000 compounds was used for sequential VS. All the database compounds in 2D, SD form were transferred into multiconformer with DS2.5. During the process of database generation, the FAST method was selected, and the maximum number of conformers generated was set to 100. The well-validated pharmacophore models include the chemical functionalities responsible for the bioactivities; therefore, the pharmacophore models were employed one by one with the fast flexible search method to screen the databases with DS2.5. The flow chart showing these sequential VS steps is illustrated in Figure 2.

Sequential VS involves the following four filters. At the first stage of VS, developed top AChE pharmacophore model was used as a 3D query to filter ZINC database compounds having low predicted AChE inhibitory activity (IC_50_) value. In order to obtain the AChE selective compounds from VS, the hits were subsequently screened by the developed BChE pharmacophore model and selected only those compounds having high BChE inhibitory activity (IC_50_) value. During the VS maximum omitted feature was set to zero and fast flexible search method was applied and other parameters were set to default.

### 2.5. ADMET Filtering

Poor pharmacokinetic properties and toxicity of the compounds are one of the main reasons for terminating the development of drug candidates [40]. In this context, we have computed Lipinski's rule of five [41] and other pharmacokinetic properties of the compounds, which includes distribution coefficient (Log *D*), computed aqueous solubility (Log *S*), polar surface area (PSA), percent human oral absorption, BBB penetration, CNS activity using ADME module of DS2.5, and QikProp module of Schrödinger software.

The rule of five suggested that a chemical compound could be an orally active drug in humans. The rule states that the most “drug-like” compounds present octanol/water partition coefficient (Log *P*), ≤ 5, molecular weight (MW) ≤ 500, and number of hydrogen-bond acceptors ≤ 10 and hydrogen-bond donors ≤ 5. Compounds violating more than one of these rules may have problems with bioavailability. Toxicity profiles of the compounds were assessed using DEREK software [42]. DEREK is a knowledge-based expert system for the qualitative prediction of toxicity. DEREK makes its predictions based on a series of rules and each rule describes the relationship between a structural feature or toxicophore and its associated toxicity. In addition to carcinogenicity, toxicological end points currently covered by the DEREK system include mutagenicity, skin sensitization, irritancy, teratogenicity, and neurotoxicity [42].

### 2.6. Docking and Protein-Ligand Interaction Analysis

Molecular docking is a computationally intensive and prominent method in drug discovery process. VS followed by docking has become one of the important methods for enhancing the efficiency in lead optimization. The benefit of docking is to identify the binding mode of ligands in the binding pocket of protein through specific key interactions and to predict the binding affinity between the protein-ligand complexes [32]. Given the crystal structure of the target, molecular docking automatically samples ligand conformations with a specified region of the protein surface. It has been successfully used for identifying active compounds by filtering out those that do not fit into the binding site [43, 44]. Plenty of crystal structures of AChE in complex with small compounds are available in the Protein Data Bank (PDB). The major differences in the CS and PAS conformations between these complexes are the orientation of F330 and Y279 residues. Among the available crystal structures of the AChE enzyme, docking studies were performed on the* Torpedo californica *AChE (*Tc*AChE) structure (PDB ID: 1EVE), considering that size and shape complementarity as well as the dual binding site nature of the donepezil was similar to xanthostigmine.* Tc*AChE has almost identical amino acid residues with the* human*AChE (*h*AChE) (PDB ID: 1B41) [45] at both the CS and PAS, apart from the substitution of F330 (*Tc*) with Y337 (human).

Genetic optimization for ligand docking (GOLD) program [46] was used for docking study and the GOLD score option was selected as the fitness function, denoted in short form as GOLD fitness score (GFS). The X-ray coordinates of donepezil bound to the active site of the AChE enzyme were used to define active site region with an active site radius of 9.5 Å, by keeping all the water within the active site. The annealing parameters of van der Waals and hydrogen-bonding interactions were considered within 4.0 Å and 2.5 Å, respectively, and other parameters were kept at the default setting. Superimposition of the docked donepezil onto the crystallographic geometry yielded RMSD of 0.55 Å, which revealed that GOLD program performed well in reproducing experimentally observed binding conformation of donepezil with a GFS of 66.79 kJ/mol.

## 3. Results

### 3.1. Pharmacophore Models Generation

In total, 10 pharmacophore models were generated for both 30 AChE and 25 BChE inhibitors from the training set in HypoGen studies with the BEST conformer generation method. The cost values, correlation coefficient (*r*), and root mean square deviation (RMSD) values together with the pharmacophore features for all of the hypotheses of AChE and BChE are listed in Tables 1 and 2, respectively. The total cost of the 10 best AChE hypotheses varied from 131.74 to 167.07, and the fixed cost value was found to be 114.68 bits, indicating good hypotheses. The null cost of top 10 AChE hypotheses was 348.99 bits, while configuration cost was 12.65 bits. The total cost value for the 10 best BChE hypothesis ranges from 112.52 to 131.94, and the fixed cost value for each of these hypotheses was 100.39; the total cost was close to the fixed cost, indicating good hypotheses. Null cost of top 10 BChE hypotheses was 167.07 bits and configuration cost was 15.18 bits. Hypo1_A was found to be the best hypotheses among 10 AChE hypotheses based on the highest correlation coefficient (0.966), the lowest RMSD value (1.045 Å), the least total cost (131.75 bits), and the highest cost difference (217.25 bits).

**Figure 3:**
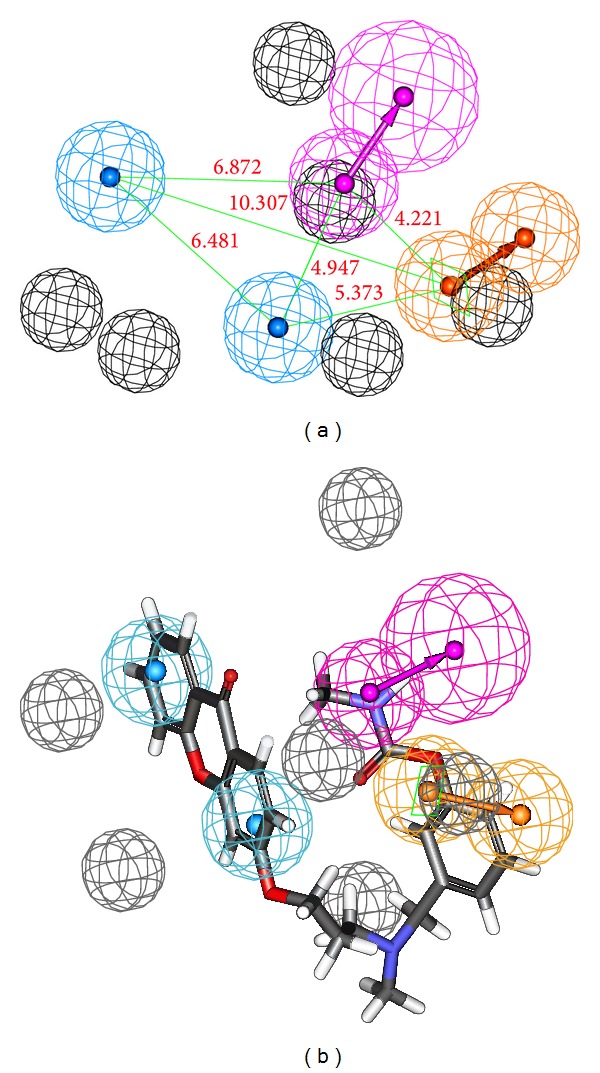
Pharmacophore model of AChEIs generated by HYPOREFINE. (a) The best HYPOREFINE model Hypo1_A. (b) Hypo1_A mapping with one of the most active compound** 1** (IC_50_ = 0.3 nM). Pharmacophore features are color-coded with light-blue for hydrophobic-aromatic feature, orange for ring aromatic feature, magenta for hydrogen-bond donor, and grey for excluded volumes.

Among 10 hypotheses generated by BChE inhibitors, Hypo1_B was characterized as the best hypothesis having the highest correlation coefficient (0.942), the lowest RMSD value (0.876 Å), the least total cost (112.52 bits), and the highest cost difference (54.55 bits). It is evident that as error, weight, and configuration costs are very low, the total cost is also low and close to the fixed cost, which implies that the correlation of generated pharmacophore model was not obtained by chance. Further, the best AChE hypothesis Hypo1_A consists of spatial arrangement of four chemical features, including one hydrogen-bond donor (HDB), two hydrophobic-aromatic (HY-AR), and one ring aromatic (RA) features along with 6 excluded volumes, as presented in Figure 3(a). The distance between the two HY-AR features was 6.481 Å, while that between one of the HY-AR and RA features was 10.307 Å and with other HY-AR and RA features was 5.373 Å. Apparently, the RA and hydrophobic interactions were essential for AChE binding, as the active site of AChE is composed of 14 conserved aromatic amino acid residues.  Figure 3(b) represents the best AChE pharmacophore model aligned with the most active AChEI with IC_50_ of 0.3 nM, which shows that pharmacophore features are mapped well to the active compound.

**Figure 4:**
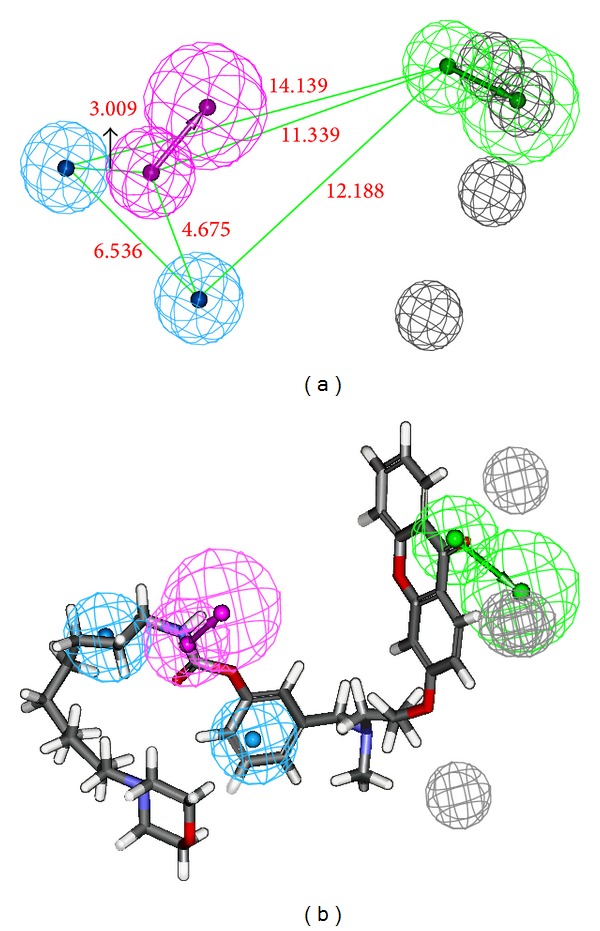
Pharmacophore model of BChE inhibitors generated by HYPOREFINE. (a) The best HYPOREFINE model Hypo1_B. (b) Hypo1_B mapping with one of the most active compound** 17** (IC_50_ = 3.3 nM). Pharmacophore features are color-coded with blue for hydrophobic feature, orange for ring aromatic feature, green for hydrogen-bond acceptor, and grey for excluded volumes.

On the other hand, the best BChE hypothesis Hypo1_B possesses four chemical features, including one hydrogen-bond acceptor (HBA), one hydrogen-bond donor (HDB), one hydrophobic-aromatic (HY-AR), and one hydrophobic aliphatic (HY-Al) features, along with 4 excluded volumes as presented in Figure 4(a). The distances between HBA and HY-AR and HBA and HY-Al features were found to be 12.188 Å and 14.139 Å, respectively, while that distance between HBD and HBA was 11.339 Å (Figure 4(a)). Further, Figure 4(b) represents the best BChE pharmacophore model aligned with the most active BChE inhibitor** 1** with IC_50_ 3.3 nM, which shows that the pharmacophoric features are mapped well to the most active inhibitor. Also, values of the correlation coefficients between the observed and calculated activities of the training set compounds occurred within the range 0.888–0.966 for AChEIs and 0.792–0.942 for BChE inhibitors. The results showed that our developed pharmacophore hypotheses were statistically significant.

### 3.2. Pharmacophore Models Validation

To verify the prediction accuracy of Hypo1_A and Hypo1_B, training set of AChE and BChE inhibitors was used and the activity of each compound in training set was predicted by regression analysis. Training set compounds of AChEIs were classified relatively into three categories based on their activity values: highly active (IC_50_ < 50 nM), moderately active (50 nM ≤ IC_50_ < 2500 nM), and low active (IC_50_ ≥ 2500 nM). In AChEIs training set, only one active compound was predicted as moderately active and two moderately active compounds are predicted as highly active. All the remaining compounds in the training set were predicted correctly by Hypo1_A. The experimental and predicted activities by Hypo1_A for 30 AChEI training set compounds are shown in Table 3. Further, to access the discriminating ability of Hypo1_B, BChE inhibitor training set compounds were classified according to their activity values: highly active (IC_50_ ≤ 100 nM), moderately active (100 nM < IC_50_ ≤ 1500 nM), and low active (IC_50_ > 1500 nM).

**Figure 5:**
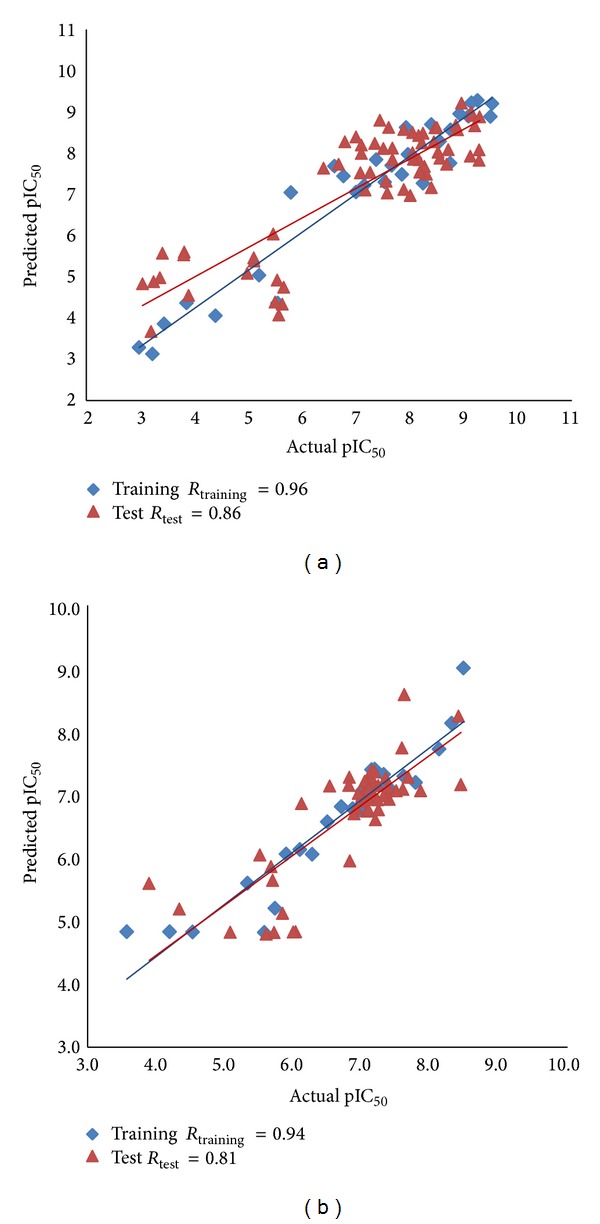
Correlation plot between the experimental and predicted activity. (a) For AChEIs by Hypo1_A for the training set and test set compounds. (b) For BChE inhibitors by Hypo1_B for the training set and test set compounds.

The predicted activities of the training set compounds by Hypo1_B along with error values and fitness scores are listed in Table 4. It suggested that Hypo1_B has good ability to predict the activity values of training set compounds. As shown in Table 4, in the training set, all 25 compounds were correctly predicted. It was clear from Tables 3 and 4 that the Hypo1_A and Hypo1_B were able to estimate the activities of compounds in their own activity ranges and the difference between the experimental and predicted activities is also minimal. Thus, error values were very less, and the fit value gives a good measure of how the defined features in Hypo1_A and Hypo1_B fit well with the pharmacophore of each compounds. Use of the quantitative pharmacophore modeling is not only to predict the activity of the training set compounds properly, but also to validate whether the model is capable of predicting the activity of external compounds of the test set series. The test set of AChEIs includes 65 compounds, while the test set of BChE inhibitors contains 51 compounds. Test sets were used to validate the best pharmacophore models, Hypo1_A for AChEIs and Hypo1_B for BChE inhibitors. All the test set compounds were prepared by the same way as that for the training set compounds. Hypo1_A was regressed against the compounds of AChEIs test set, while the Hypo1_B was regressed against the test set of BChE inhibitors, yielding a correlation coefficient of 0.86 for AChEIs test set and 0.81 for BChE inhibitors test set, respectively. The results are presented in Figures 5(a) and 5(b) for AChE and BChE inhibitors test set, respectively, which suggested a good correlation between the experimental and predicted activities. These statistically significant results provide confidence on our AChE (Hypo1_A) and BChE (Hypo1_B) pharmacophore models.

**Figure 6:**
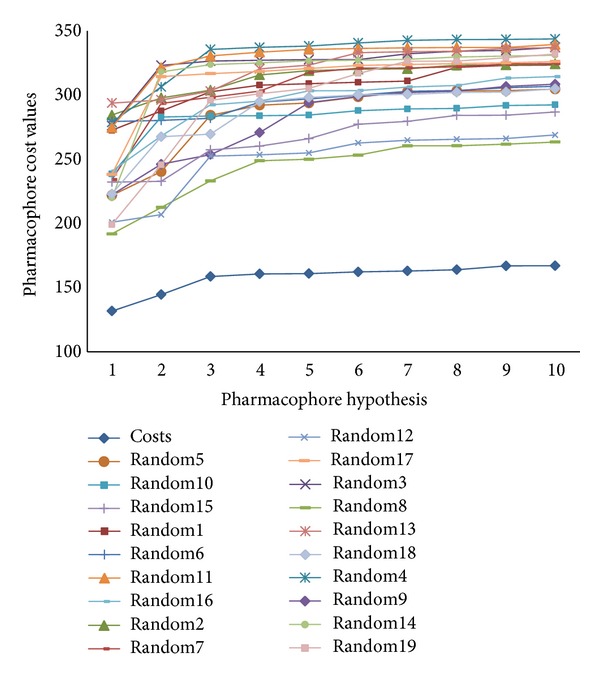
The difference in costs between HYPOGEN runs and the scrambled runs for Hypo1_A. The 95% confidence level was selected.

**Figure 7:**
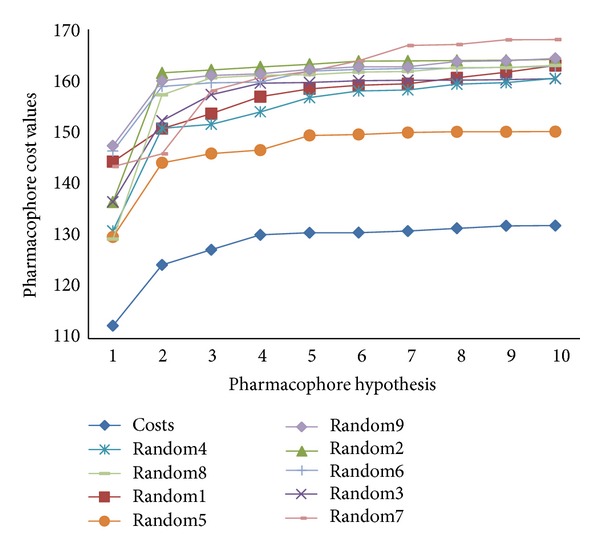
The difference in costs between HYPOGEN runs and the scrambled runs for Hypo1_B. The 90% confidence level was selected.

To further confirm the statistical significance of the developed pharmacophore models Hypo1_A and Hypo1_B, Fischer's randomization test was performed by Cat-Scramble program implemented in DS2.5. Each compound in the training set was randomly reassigned activity values and subsequently generated hypotheses; spreadsheets were obtained with the randomized activity data. For Cat-Scramble validation of Hypo1_A, confidence level of 95% was selected, and thus 19 spreadsheets were generated, while confidence level of 90% was selected, which generated 9 spreadsheets for the validation of Hypo1_B by Cat-Scramble program. All spreadsheets were used to construct hypotheses using exactly the same conditions as used in generating the original pharmacophore hypotheses. These results are reported in Tables 5 and 6, respectively, and it was found that none of the 19 resulting hypotheses for AChEIs and 9 resulting hypotheses for BChE inhibitors after randomization has a better cost value compared with the Hypo1_A and Hypo1_B pharmacophore hypotheses. Thus, this cross-validation further confirmed the correlation of structures and experimental activities in the training set and provided us with strong confidence on Hypo1_A and Hypo1_B. The total costs of pharmacophore models obtained in the 19 HYPOREFINE runs for Hypo1_A and 9 HYPOREFINE runs for Hypo1_B as well as the original HYPOREFINE run are presented in Figures 6 and 7, respectively.

### 3.3. Sequential Virtual Screening

We adopted a sequential VS procedure, wherein the pharmacophore based VS was followed by predicted activity prefiltration, molecular docking, and further ADMET screening. Initially, Hypo1_A was used as 3D query to screen the ZINC database, which consists of 45,00,000 compounds and the predicted bioactivity of the retrieved hits was also obtained. A hit list of 151276 compounds matching the entire critical pharmacophore model was obtained.

Thereafter the 3D quantitative pharmacophore Hypo1_A was used to estimate the AChE inhibitory activity of the initial hits, and it was found that a total of** 1866** hits had predicted IC_50_ values less than 100 nM (IC_50_ ≤ 100 nM) as presented in Figure 1. To sample a sufficient chemical space and increase hit rate, hits with predicted IC_50_ ≤  100 nM were considered as active new hits. The 1866 hits were subsequently screened by the developed best pharmacophore model Hypo1_B of BChE by the ligand pharmacophore mapping module of DS2.5. The flow chart showing these sequential VS steps is illustrated in Figure 1. Out of the 1866 hits, a total of 1164 compounds satisfied all the critical features in Hypo1_B. To obtain the AChE selective compounds from VS, we applied a reverse screening approach and selected only those compounds having BChE inhibitory activity predicted by Hypo1_B which were greater than or equal to 10,000 nM (IC_50_ ≥ 10,000 nM).

### 3.4. ADMET Filtering

The assessment of ADMET (absorption, distribution, metabolism, excretion, and toxicity) properties of the compounds at the early stages of drug discovery is a very important indicator for selecting the compounds for further studies [47]. The drug-likeness, other physicochemical properties, and toxicity analysis were performed of 706 compounds obtained from the dual pharmacophore screening (Figure 2). The physicochemical properties linked with compounds that have good blood-brain barrier (BBB) penetration, optimum oral bioavailability, less or no toxicity, and also optimum solubility are the significant filters for selecting CNS active compounds and there is a need for compounds with good pharmacokinetic properties [48]. After carefully analyzing the ADMET properties of 706 compounds obtained from the dual pharmacophore based screening, we finally considered 105 hits, which are not showing any toxicity or minor toxicity predicted by the DEREK, also having good pharmacokinetic parameter and CNS activity was proceeded for further evaluation by molecular docking analysis.

### 3.5. Molecular Docking Analysis

In order to further filter the retrieved 105 hits, molecular docking analysis was performed at the dual binding site of AChE enzyme using GOLD program. Docking results were reported as the highest scoring pose for each compound and also on the basis of their ability to form favorable interactions within the active site of the AChE enzyme. Based on the GFS function finally 18 compounds were sorted for further* in vivo* studies. All of the hit compounds possessed the good fit value with Hypo1_A and the GFS higher or equal to 55 kJ/mol (GFS ≥ 55 kJ/mol). The hit compounds showed very good interactions with the critical residues of PAS W279 and AS W84, respectively.

## 4. Discussion

In this work, we first generated specific quantitative 3D-pharmacophore models of AChE and BChE inhibitors to identify the critical chemical features for AChE and BChE inhibitors. The generated 3D pharmacophoric models were ranked based on their lowest total cost, highest cost difference between null cost, and total cost and the configuration cost of hypotheses. There is no relationship between the experimental and predicted biological activities for the calculation of null hypotheses cost. The configuration cost should not exceed the recommended value of 15 and could assure the entire conformation space sampled during the pharmacophore generation. The root mean square differences (RMSD) between the predicted and experimental biological activities of the training set molecules are proportional to the error cost. Cost difference between the null hypotheses cot and the total cost is the highest for Hypo1_A (Table 5) and Hypo1_B (Table 6), which signifies that the correlation between the fit values and experimental activities is not a random occurrence. The 30 AChE inhibitors of training set were mapped on the best AChE pharmacophore Hypo1_A among the 10 generated hypotheses based on the highest correlation coefficient (0.966) and the lowest RMSD value (1.045 Å), which shows a good correlation between the experimental activities and predicted fit values. Similarly, 25 training set BChE inhibitors were aligned on the best BChE pharmacophore Hypo1_B among the 10 generated hypotheses based on the highest correlation coefficient (0.942) and the lowest RMSD value (0.876 Å). The best AChE pharmacophore model, Hypo1_A, consists of four chemical features: one HDB, two HY-AR, and one RA along with 6 excluded volumes. While, the best BChE pharmacophore model, Hypo1_B, possesses four chemical features, including one HBA, one HDB, one HY-AR, and one HY-Al features, along with 4 excluded volumes. The two HY-AR features are properly mapped on the aromatic rings of the xanthone moiety of the most active AChEI compound** 1** (Figure 3(b)). This analysis was also supported by docking study in which the compound** 1** occupies the hydrophobic region in AChE enzyme formed by the F300 and F331 residues reported by Rampa et al. [10]. Also, the docked pose of compound** 1** attains almost similar conformation in the active site of AChE as it is mapped on Hypo1_A (Figure 3(b)). This provides us with an additional confidence to the developed AChE pharmacophore model Hypo1_A.

The best Pharmacophore models Hypo1_A and Hypo1_B were further validated by Fischer's randomization test and test set prediction. Results of Fischer's randomization test for Hypo1_A (Figure 6) and Hypo1_B (Figure 7) clearly show that the Hypo1_A and Hypo1_B are not generated by chance, because its statistics are far more superior to all random hypotheses. The test set prediction suggests a good correlation between the experimental and predicted activities for both Hypo1_A (correlation coefficient *r* = 0.86) and Hypo1_B (correlation coefficient *r* = 0.81) indicates the good prediction ability of both models. We performed virtual screening (VS) on Zinc database to identify potent dual binding site and selective AChE inhibitors by combining dual pharmacophore models, ADMET screening and finally by docking analysis to examine important interactions responsible for binding to AChE. Molecular docking acted as an additional tool for pharmacophore based virtual screening, the concurrent use of which is believed to make the discovery of potent, selective, and dual binding site AChE inhibitors more efficient.

**Figure 8:**
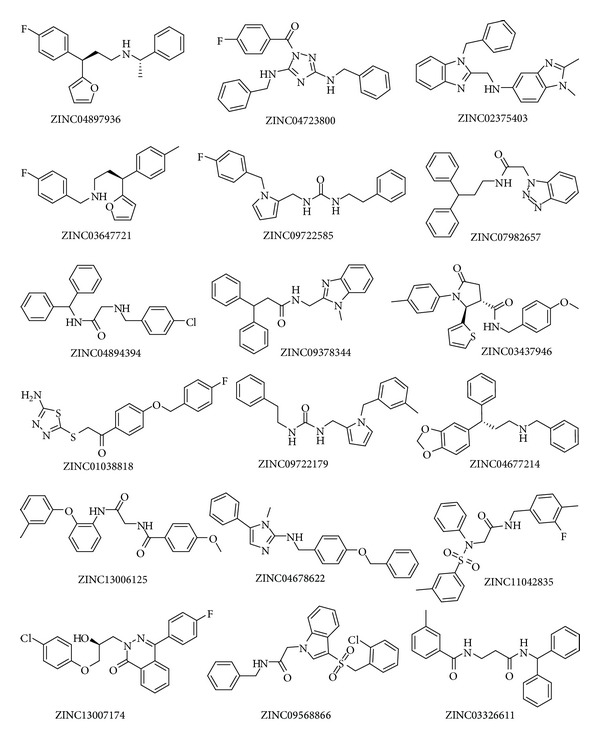
Chemical structures of 18 VS compounds from ZINC database together with their ZINC codes.

**Figure 9:**
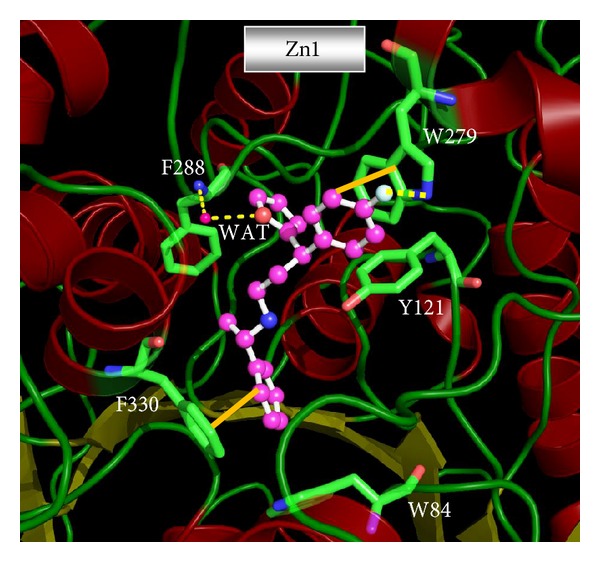
Molecular docking derived binding pose of the VS lead compounds Zn1 in the dual binding site (CS and PAS) of AChE enzyme. The inhibitor is shown as ball and stick model in the surface representation of the enzyme. Water compounds are shown as red dotted spheres. GOLD software was used to derive the binding mode and the picture was generated from PyMOL software.

**Figure 10:**
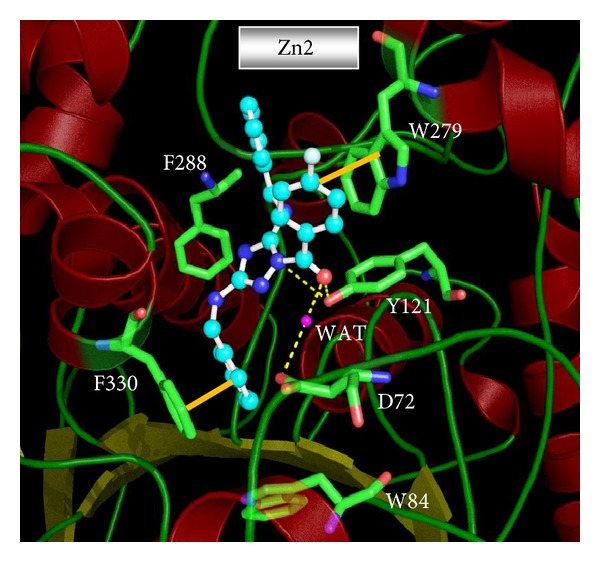
Molecular docking derived binding pose of the VS lead compounds Zn2 in the dual binding site (CS and PAS) of AChE enzyme. The inhibitor is shown as ball and stick model in the surface representation of the enzyme. Water compounds are shown as red dotted spheres. GOLD software was used to derive the binding mode and the picture was generated from PyMOL software.

We identified the 18 promising dual binding site AChE inhibitors with all favorable drug-like properties from ZINC database. The predicted AChE and BChE inhibitory activity IC_50_ (nM) and calculated ADMET properties of these 18 compounds and their corresponding GFS values are given in Table 7. The structure of these 18 VS compounds is presented in Figure 8, along with their ZINC codes. The binding orientation of the top two hit compounds from VS, ZINC04897936 (Zn1) (AChE IC_50_ = 17.76 nM, BChE IC_50_ = 15,197.5 nM), and ZINC04723800 (Zn2) (AChE IC_50_ = 22.56 nM, BChE IC_50_ = 16,502.8 nM) is shown in Figures 9 and 10, respectively. The best VS compound Zn1 forms two *π*-*π* stacking interactions (i) between the benzene ring of Zn1 and aromatic ring of F330 (AS) in AChE enzyme, and (ii) between fluorobenzene ring of Zn1 stacked against the indole ring of W279 at the PAS of the AChE enzyme (Figure 9). Apart from that, Zn1 also shows two hydrogen bonds with active site of AChE enzyme; one water mediated hydrogen bond was formed between the oxygen atom of the pyrrole ring of Zn1 and amide (NH) group of the F288. The second hydrogen bond was observed between the fluorine atom of Zn1 and the hydrogen atom attached with the nitrogen atom of indole ring in the PAS of the AChE enzyme, which further confirms a strong binding between Zn1 and PAS of the AChE enzyme.

It was already known that PAS of the AChE was involved in increasing the aggregation rate of the A*β*; hence strong binding with the PAS will stop the aggregation rate and provide the disease modifying effect along with the symptomatic benefit. The top scored docking pose of the second most active VS compound Zn2 also shows the similar binding trends in docking as shown in Figure 10. The Zn2 form a *π*-*π* stacking interaction with F330 at the bottom of the CS and to reach the PAS for *π*-*π* stacking interaction with W279. Compound also showed strong bonding at the mid of gorge through two hydrogen bonds. One hydrogen bond was formed between carbonyl group (C=O) of the Zn2 and hydroxyl group of Y121. Besides this, second water mediated hydrogen bond was observed between carbonyl group (C=O) of the Zn2 and carboxylic acid group of D72, which suggests that the water mediated interactions are present in AChE enzyme; similar interactions were observed in donepezil and also our previously published VS AChE active compounds [20].

**Figure 11:**
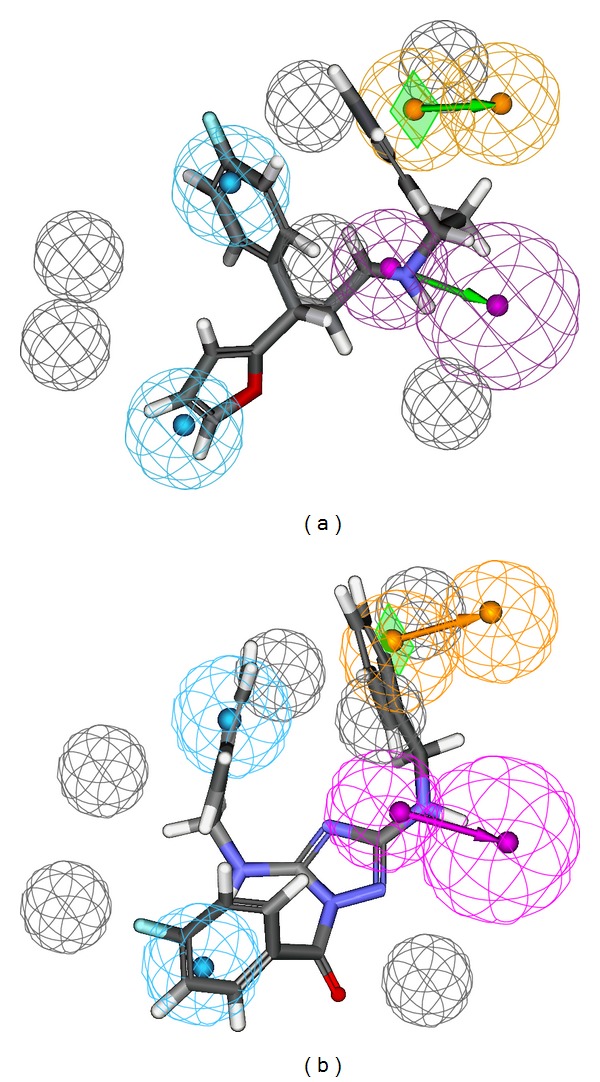
The best HYPOREFINE model Hypo1_A mapping with one of the most active VS compound Zn1 (IC_50_ = 17.76 nM) (a). Hypo1_A mapping with the second most active VS compound Zn2 (IC_50_ = 22.56 nM) (b). Pharmacophore features are color-coded with light-blue for hydrophobic-aromatic feature, orange for ring aromatic feature, magenta for hydrogen-bond donor, and grey for excluded volumes.

It is known that the active site of the AChE is composed of hydrophobic residues and more over the F330 and W279 showing dynamical motion, which is confirmed by the crystal structures of AChE bound with different ligands. The donepezil bound crystal structure F330 shows an open conformation, while the tacrine bound crystal structure F330 has a closed conformation. In our present docking study, open conformation of F330 was observed with the Zn1 and Zn2 compounds. The W279 also attain the similar conformation as in donepezil bound crystal structure (1EVE), which is in accordance with the Zn1 and Zn2 docked compounds (Figures 9 and 10). The alignment of these top two hits with the Hypo1_A is presented in Figures 11(a) and 11(b), respectively. The details of the selected 18 VS compounds are presented in Table 7. These compounds will be shifted to* in vitro* studies for their inhibitory potency evaluation.

## 5. Conclusions

In this study, we have reported integrated pharmacophore based sequential virtual screening protocol to identify dual binding site and selective AChEIs from the ZINC database. The AChE and BChE specific 3D-pharmacophore models were generated using 30 training set compounds for AChEI and 26 training set compounds for BChE inhibitor. The best quantitative pharmacophore hypotheses, Hypo1_A and Hypo1_B, were characterized by their high cost difference, high correlation, low RMSD, and configuration cost values. Both pharmacophore models were well validated to be of high predictability for estimating the activities over a variety of compounds and evaluating how well diverse compounds can be mapped onto the pharmacophore before conducting any further experimental study. Hypo1_A consists of one hydrogen-bond donor (HBD), two hydrophobic-aromatic (HY-AR), and one ring aromatic (RA) features along with 6 excluded volumes. The mapping study of pharmacophore demonstrated that xanthone could be the main scaffold on which substitutions led to increase the AChE inhibitory activity. The other ring aromatic feature from the xanthone seems to be relevant to influence the inhibitory activity. Hypo1_B consists of one hydrogen-bond acceptor (HBA), one hydrogen-bond donor (HDB), one hydrophobic-aromatic (HY-AR), and one hydrophobic aliphatic (HY-Al) features along with 4 excluded volumes. Molecular docking and ADMET analysis acted as an additional tool for pharmacophore based VS. From the overall sequential VS analyses, we identified 18 promising hits from ZINC database. Moreover, the leads obtained from VS have all the properties required by drug-like compounds. Molecular docking studies on Zn1 and Zn2 compounds allowed an in-depth analysis and interpretation of the dual binding site (AS and PAS) interactions of the inhibitors with the AChE enzyme.

## Figures and Tables

**Table 1:** Statistical parameters of top 10 pharmacophores for AChEIs generated by the HypoGen.

Hypo. number	Total cost	Cost diff.	RMSD (Å)	Correlation (*r*)	Features
Hypo1_A	131.75	217.25	1.046	0.9664	HBD, 2HY-AR, RA, E6
Hypo2_A	144.56	204.43	1.389	0.9399	HBD, 2HY-AR, RA, E6
Hypo3_A	158.68	190.31	1.697	0.9089	HBD, HY-Al, HY-AR, RA, E4
Hypo4_A	160.67	188.32	1.739	0.9039	HBD, 2HY-AR, RA, E5
Hypo5_A	160.92	188.07	1.755	0.9021	HBD, HY-Al, HY-AR, RA, E2
Hypo6_A	162.29	186.71	1.740	0.9041	HBD, HY-AR, RA, E4
Hypo7_A	162.96	186.03	1.785	0.8985	HBD, HY-AR, RA, E5
Hypo8_A	163.94	185.06	1.809	0.8957	HBD, HY-AR, RA, E4
Hypo9_A	166.92	182.07	1.859	0.8895	HBD, HY-AR, RA, E4
Hypo10_A	167.07	181.92	1.864	0.8888	HBD, HY-AR, RA, E4

Null cost of top 10 score hypotheses is 348.99 bits; fixed cost is 114.68 bits; configuration cost is 12.65 bits. Abbreviations used for features are as follows: HBD: H-bond donor; HY-AR: hydrophobic aromatic; RA: ring aromatic; HY: hydrophobic; E: excluded volumes.

**Table 2:** Statistical parameters of top 10 pharmacophore models for BChE inhibitors generated by the HypoGen.

Hypo. number	Total cost	Cost diff.	RMSD (Å)	Correlation (*r*)	Features
Hypo1_B	112.52	54.55	0.876	0.9429	HBA, HBD, HY-Al, HY-AR, 4E
Hypo2_B	124.32	42.75	1.374	0.8464	HBA, HBD, HY-Al, RA
Hypo3_B	127.26	39.80	1.464	0.8228	HBA, HBD, HY-Al, HY-AR
Hypo4_B	130.14	36.92	1.538	0.8023	HBA, HBD, HY-Al, 2RA
Hypo5_B	130.55	36.51	1.553	0.7978	HBA, HBD, HY-Al, HY-AR, 2E
Hypo6_B	130.56	36.50	1.551	0.7988	HBA, HBD, HY-Al, HY-AR
Hypo7_B	130.89	36.18	1.560	0.7959	HBA, HBD, HY-Al, RA, 2E
Hypo8_B	131.41	35.65	1.569	0.7934	HBA, HBD, HY-Al, HY-AR
Hypo9_B	131.89	35.18	1.582	0.7895	HBA, HBD, HY-Al, HY-AR
Hypo10_B	131.94	35.12	1.573	0.7928	HBA, HBD, HY-Al, HY-AR, E

*Null cost of top 10 score hypotheses is 167.06 bits; fixed cost is 100.39 bits; configuration cost is 15.18 bits.

**Table 3:** Experimental and predicted IC_50_ values of the AChEIs in training set.

S. number	Actual IC_50 _(nM)	Predicted IC_50 _(nM)	Error	Fit value	Exptl. scale	Predicted scale
1.	0.30	0.64	2.14	6.70	+++	+++
2.	0.32	1.33	4.16	6.38	+++	+++
3.	0.56	0.53	−1.05	6.78	+++	+++
4.	0.72	0.61	−1.19	6.73	+++	+++
5.	0.82	1.30	1.59	6.39	+++	+++
6.	1.20	1.13	−1.06	6.45	+++	+++
7.	1.76	2.76	1.57	6.07	+++	+++
8.	1.80	17.81	9.89	5.26	+++	+++
9.	2.80	5.30	1.89	5.78	++	+++
10.	4.0	2.05	−1.95	6.20	+++	+++
11.	5.7	55.01	9.65	4.77	+++	++
12.	8.0	13.72	1.72	5.37	+++	+++
13.	10.9	10.81	−1.01	5.47	+++	+++
14.	11.7	2.42	−4.84	6.13	+++	+++
15.	14.0	33.28	2.38	4.99	+++	+++
16.	21.8	20.18	−1.08	5.20	+++	+++
17.	29.3	51.36	1.75	4.80	+++	++
18.	42	14.69	−2.86	5.34	+++	+++
19.	70	61.08	−1.15	4.72	++	++
20.	100	89.73	−1.11	4.56	++	++
21.	170	36.87	−4.61	4.94	++	+++
22.	250	21.11	−11.84	5.18	++	+++
23.	1,610	92.01	−17.50	4.54	++	++
24.	2,820	43470.30	15.42	1.87	+	+
25.	6,250	9215.51	1.47	2.54	+	+
26.	40,000	88732.50	2.22	1.56	+	+
27.	138,000	43529.00	−3.17	1.87	+	+
28.	360,000	139736.00	−2.58	1.36	+	+
29.	594,000	748966.00	1.26	0.63	+	+
30.	1,050,000	522681.00	−2.01	0.79	+	+

**Table 4:** Experimental and predicted IC_50_ values of BChE inhibitors in the training set.

S. number	Actual IC_50_ (nM)	Estimate IC_50_ (nM)	Error	Fit value	Exptl. scale	Predicted scale
17.	3.3	1	−3.3	9.53	+++	+++
31.	4.9	7.4	1.5	8.66	+++	+++
32.	7.5	20	2.7	8.22	+++	+++
2.	16	65	4	7.72	+++	+++
33.	24.5	53	2.1	7.81	+++	+++
34.	38.3	94	2.4	7.56	+++	+++
35.	41.5	78	1.9	7.64	+++	+++
1.	48	48	−1	7.85	+++	+++
36.	65	39	−1.7	7.94	+++	+++
37.	72	41	−1.8	7.92	+++	+++
8.	81	100	1.3	7.52	+++	+++
38.	100	84	−1.2	7.6	+++	+++
4.	110	160	1.5	7.32	++	++
39.	136	160	1.2	7.32	++	++
9.	200	160	−1.3	7.34	++	++
40.	320	250	−1.3	7.14	++	++
41.	529	870	1.6	6.59	++	++
24.	800	640	−1.2	6.72	++	++
42.	1270	850	−1.5	6.6	++	++
26.	1800	5100	2.8	5.82	+	+
25.	2600	16000	5.9	5.34	+	+
43.	4650	2600	−1.8	6.11	+	+
44.	29500	14000	−2.1	5.38	+	+
29.	64000	15000	−4.3	5.36	+	+
45.	269000	15000	−18	5.35	+	+

**Table 5:** Results of Fischer's randomization test for Hypo1_A at 95% using Cat-Scramble implemented in DS2.5 software.

Validation number	Total cost	Null cost	Cost diff.	Correlation (*r*)
Hypo1_A	121.22	309.61	188.38	0.9588

Results for scrambled				
Trial 1	247.12	309.61	62.49	0.6452
Trial 2	216.08	309.61	93.53	0.8326
Trial 3	208.01	309.61	101.59	0.8324
Trial 4	223.79	309.61	76.82	0.7378
Trial 5	232.78	309.61	76.82	0.7090
Trial 6	257.60	309.61	52.01	0.6158
Trial 7	205.35	309.61	104.26	0.7857
Trial 8	207.48	309.61	102.13	0.7614
Trial 9	223.63	309.61	85.98	0.7407
Trial 10	216.63	309.61	92.98	0.7352
Trial 11	259.98	309.61	49.62	0.5540
Trial 12	157.19	309.61	152.42	0.9044
Trial 13	229.49	309.61	80.12	0.7024
Trial 14	209.51	309.61	100.10	0.7530
Trial 15	206.51	309.61	103.10	0.7601
Trial 16	239.05	309.61	104.21	0.7536
Trial 17	205.40	309.61	104.21	0.7917
Trial 18	233.94	309.61	75.67	0.6884
Trial 19	218.95	309.61	90.66	0.8098

**Table 6:** Results of Fischer's randomization test for Hypo1_B at 90% using Cat-Scramble implemented in DS2.5 software.

Validation number	Total cost	Null cost	Cost diff.	Correlation (*r*)
Hypo1_B	112.52	167.07	54.55	0.9429

Results for scrambled				
Trial 1	144.34	167.07	22.73	0.6456
Trial 2	136.53	167.07	30.54	0.7953
Trial 3	136.55	167.07	30.52	0.7425
Trial 4	130.93	167.07	36.14	0.7851
Trial 5	129.73	167.07	37.34	0.8420
Trial 6	146.39	167.07	20.68	0.6503
Trial 7	143.40	167.07	23.67	0.7184
Trial 8	129.32	167.07	37.75	0.8321
Trial 9	147.42	167.07	19.65	0.6229

**Table 7:** The binding scores, ADMET properties, and predicted AChE inhibitory IC_50_ (nM) values of the top ranking conformers of the representative hits.

Number	ZINC codes	CNS	PSA	*A*log⁡*P*	log⁡*D*	%HOA	Solu.	Derek∗	IC_50_ (nM) AChE	IC_50_ (nM) BChE	GOLD docking
1	ZINC04897936	2	18.77	4.78	3.60	100	−5.42	HERG	17.76	15197.5	58.43
2	ZINC04723800	0	70.71	4.35	4.35	100	−6.15	Nothing	22.56	16502.8	79.50
3	ZINC02375403	0	43.08	4.70	4.72	100	−6.45	Nothing	25.12	15648.3	73.16
4	ZINC03647721	2	19.32	4.88	3.77	100	−5.48	HERG	27.80	14942.8	66.23
5	ZINC09722585	0	47.10	4.02	4.02	100	−4.30	Nothing	28.02	15216.4	72.85
6	ZINC07982657	0	57.79	4.35	4.35	100	−5.29	Nothing	32.40	16209.0	72.27
7	ZINC04894394	1	40.68	4.28	4.16	100	−4.89	HERG	40.57	11729.1	70.81
8	ZINC09378344	0	43.04	4.31	4.32	100	−5.41	Nothing	48.08	15387.3	71.29
9	ZINC03437946	0	68.60	3.82	3.82	100	−4.79	Skin	49.23	15111.7	72.77
10	ZINC01038818	0	80.38	3.66	3.66	100	−5.74	Nothing	50.66	14830.6	74.69
11	ZINC09722179	0	42.58	4.30	4.30	100	−4.39	Nothing	53.62	14938.7	71.56
12	ZINC04677214	1	31.96	4.78	4.02	100	−5.56	HERG	57.70	14837.7	59.01
13	ZINC13006125	0	82.30	3.70	3.70	100	−4.44	Skin	62.18	14811.3	71.13
14	ZINC04678622	0	33.12	4.76	4.95	100	−5.73	Nothing	66.28	20416.1	70.05
15	ZINC11042835	0	62.81	4.34	4.34	100	−5.25	Nothing	66.42	14922.5	62.19
16	ZINC13007174	0	53.53	4.70	4.70	100	−5.41	Nothing	69.02	14835.2	68.24
17	ZINC09568866	0	73.19	4.74	4.74	100	−5.84	Nothing	79.39	14974.8	77.15
18	ZINC03326611	0	68.44	4.03	4.03	100	−4.42	Nothing	82.54	15048.3	69.16

*HERG: HERG channel inhibition, Nothing: no toxicity, and Skin: skin sensitization.
